# Evaluation of DNA extraction kits for long-read shotgun metagenomics using Oxford Nanopore sequencing for rapid taxonomic and antimicrobial resistance detection

**DOI:** 10.1038/s41598-024-80660-3

**Published:** 2024-11-27

**Authors:** Srinithi Purushothaman, Marco Meola, Tim Roloff, Ashley M. Rooney, Adrian Egli

**Affiliations:** Institute of Medical Microbiology, University of Zurich, Gloriastrasse 30, Zurich, 8006 Switzerland

**Keywords:** Metagenomics, Long-read, Taxonomy, DNA extraction, Protocol, ONT, Antimicrobial resistance., Clinical microbiology, Metagenomics, Microbiology, Microbial communities, Infectious-disease diagnostics

## Abstract

During a bacterial infection or colonization, the detection of antimicrobial resistance (AMR) is critical, but slow due to culture-based approaches for clinical and screening samples. Culture-based phenotypic AMR detection and confirmation require up to 72 hours (h) or even weeks for slow-growing bacteria. Direct shotgun metagenomics by long-read sequencing using Oxford Nanopore Technologies (ONT) may reduce the time for bacterial species and AMR gene identification. However, screening swabs for metagenomics is complex due to the range of Gram-negative and -positive bacteria, diverse AMR genes, and host DNA present in the samples. Therefore, DNA extraction is a critical initial step. We aimed to compare the performance of different DNA extraction protocols for ONT applications to reliably identify species and AMR genes using a shotgun long-read metagenomic approach. We included three different sample types: ZymoBIOMICS Microbial Community Standard, an in-house mock community of ESKAPE pathogens including *Enterococcus faecium*, *Staphylococcus aureus*, *Klebsiella pneumoniae*, *Acinetobacter baumannii*, *Pseudomonas aeruginosa*, and *Escherichia coli (*ESKAPE Mock*)*, and anonymized clinical swab samples. We processed all sample types with four different DNA extraction kits utilizing different lysis (enzymatic vs. mechanical) and purification (spin-column vs. magnetic beads) methods. We used kits from Qiagen (QIAamp DNA Mini and QIAamp PowerFecal Pro DNA) and Promega (Maxwell RSC Cultured Cells and Maxwell RSC Buccal Swab DNA). After extraction, samples were subject to the Rapid Barcoding Kit (RBK004) for library preparation followed by sequencing on the GridION with R9.4.1 flow cells. The fast5 files were base called to fastq files using Guppy in High Accuracy (HAC) mode with the inbuilt MinKNOW software. Raw read quality was assessed using NanoPlot and human reads were removed using Minimap2 alignment against the Hg38 genome. Taxonomy identification was performed on the raw reads using Kraken2 and on assembled contigs using Minimap2. The AMR genes were identified using Minimap2 with alignment against the CARD database on both the raw reads and assembled contigs. We identified all bacterial species present in the Zymo Mock Community (8/8) and ESKAPE Mock (6/6) with Qiagen PowerFecal Pro DNA kit (chemical and mechanical lysis) at read and assembly levels. Enzymatic lysis retrieved fewer aligned bases for the Gram-positive species (*Staphylococcus aureus* and *Enterococcus faecium*) from the ESKAPE Mock on the assembly level compared to the mechanical lysis. We detected the AMR genes from Gram-negative and -positive species in the ESKAPE Mock with the QIAamp PowerFecal Pro DNA kit on reads level with a maximum median time of 1.9 h of sequencing. Long-read metagenomics with ONT may reduce the turnaround time in screening for AMR genes. Currently, the QIAamp PowerFecal Pro DNA kit (chemical and mechanical lysis) for DNA extraction along with the Rapid Barcoding Kit for the ONT sequencing captured the best taxonomy and AMR identification for our specific use case.

**Supplementary Information:**

The online version contains supplementary material available at 10.1038/s41598-024-80660-3.

## Introduction

Shotgun metagenomics has been widely used to characterize microbial populations present across various environments^1,2^. Recently, it gained importance for clinical use cases, e.g., for pathogen identification, owing to its culture-independent manner to determine the microorganisms present in clinical scenarios. These include syndromal settings with a broad range of common infections such as meningitis, encephalitis, sepsis, diarrhea, and lower respiratory infections^3–6^. In current microbiological diagnostic practice, culture-based methods are considered as the reference standard for pathogen identification and determination of antibiotic susceptibility profiles^7,8^. However, one of the major drawbacks of using a culture-based microbiological diagnostic approach is the long turnaround time (TAT) of around 72 h, from sample collection to actionable insights^9,10^ as well as culture-negative samples e.g., due to prior antibiotic treatment. Thereby, treatment adaptations are substantially delayed and may result in an untargeted and non-effective or suboptimal treatment.

This critical time delay could be overcome by directly sequencing the sample using a rapid shotgun metagenomic technique. This requires a suitable and fast sequencing platform such as the long-read Oxford Nanopore Technologies (ONT) sequencer. ONT sequencing enables real-time analysis of the sequenced data with substantially reduced TAT from days to hours^11–13^. Thereby, fast and actionable results can be generated for clinical management. A few proof-of-concept studies demonstrated the application of ONT-based shotgun metagenomics in clinical diagnosis directly from various patient sample types such as tissues, stool, urine, cerebrospinal fluid, and sputum^14–18^. Several studies are implementing full-length 16S for bacterial identification in clinics^19–21^ for pathogen identification. However, there are reasons to prefer shotgun metagenomics over 16S sequencing. Some of the limitations of full-length 16S sequencing include PCR amplification bias^22^ and the inability to detect antimicrobial resistance (AMR) genes^23^. Rectal and nasopharyngeal swabs are widely used for screening AMR bacteria colonization or infection^24,25^. These screening swabs may collect a wide range of Gram-positive and -negative bacteria potentially harboring different classes of AMR genes, along with host material. Thus, the processing of these complex clinical samples should be carried out with utmost care. Biases introduced during pre-analytics can affect the sequencing outcome and might mislead the diagnostic conclusions^26^. Standardization and selection of optimum sample storage conditions^27–29^, DNA extraction methods^30–33^, library preparation for sequencing^34,35^, and a suitable bioinformatics pipeline for data analysis^36–38^ increase the quality and reliability of shotgun metagenomics in clinical microbiology.

Among these steps, DNA extraction from the microbial cells remains one of the most critical pre-analytical factors influencing the sequencing outcome because the extracted DNA must capture the bacterial community without any gram bias^39,40^. Although studies are comparing different DNA extraction kits from clinical samples such as stool, vaginal swabs, and urine samples for metagenomic studies^41–44^ utilizing short-read technology, very few evaluation studies are available for the long-read ONT sequencing approach^45,46^.

Our evaluation study aimed to compare different DNA extraction methods to ensure the best quality for subsequent ONT sequencing in a rapid shotgun metagenomic approach using three different sample types. We included four different automated DNA extraction kits involving enzymatic and mechanical lysis and studied the impact on sequencing quality, taxonomy, and AMR gene identification.

## Materials and methods

### Preparation of samples used for evaluation

**Fig. 1:**
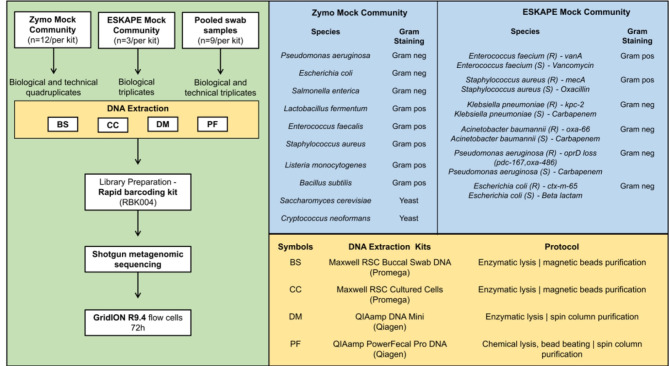
Experimental study design. The green box (left) shows the experimental workflow. The blue box (top right) outlines the composition of the two mock communities: the Zymo Mock Community and the *in-house* ESKAPE Mock Community. The yellow box (lower right) represents the four different DNA extraction kits used. Gram neg, Gram-negative; Gram pos, Gram-positive. R - antimicrobial resistant strain, and S - antimicrobial sensitive strain. The number of tested samples is shown in the green box (left). For the Zymo Mock Community (*n* = 12), this includes three technical replicates repeated across four sequencing runs (biological replicates). For the ESKAPE Mock (*n* = 3), the data represents three sequencing runs without technical replicates. The pooled swab samples (*n* = 9) include three technical replicates repeated across three sequencing runs (biological replicates).

We used three microbial community compositions: (i) ZymoBIOMICS Microbial Community Standard, (ii) an ESKAPE pathogens Mock Community^47^, and (iii) anonymized and pooled clinical eSwab solutions from rectal and nasopharyngeal swabs (Fig. 1).

We used the ZymoBIOMICS Microbial Community Standard (D6300, hereafter referred to as Zymo Mock Community) as per the manufacturer’s protocol. We extracted the DNA directly from 75µL of the thawed mock solution. The Zymo Mock Community has a total cell concentration of ~ 1.4 × 10^10^ cells/mL. The sequencing run was repeated four times, each run with three technical replicates (*n* = 12).

In addition, we prepared an ESKAPE Pathogens Mock Community (hereafter referred to as ESKAPE Mock) comprising sensitive and resistant isolates. An appendix with a description of the AMR genes is provided in the supplementary. The ESKAPE isolates were collected from routine diagnostics at the University Hospital of Basel. The ESKAPE isolates were previously characterized using short-read Illumina(150PE) sequencing, as a part of the routine diagnostics protocol with a minimum of 40x read depth for each isolate, considered as reference genomes. The Illumina libraries were prepared using Nexteraflex and sequenced on the NextSeq and Illumina reference genomes obtained as mentioned in the previous work of Seth-Smith et al.^34^ We cultured each of these ESKAPE strains individually in Mueller Hinton broth at 37°C in a shaking incubator. After the isolates reached an OD of 0.6, 200 µl from each of the individual cultures was mixed, to a final volume of 2.4mL. Then, we centrifuged the ESKAPE mock solution at 5000 g for 15 min and used the pellet for DNA extraction. In parallel, we inoculated 50 µl from each of the ESKAPE strains culture solutions and calculated the Colony Forming Units (CFU) per mL, where each strain had a mean of 10^8^ CFU/mL. The sequencing run is repeated three times, without technical replicates (*n* = 3).

For swab samples, we randomly collected, pooled, and anonymized 30 eSwab (Copan’s Liquid Amies Elution Swab) solutions consisting of clinical rectal and nasopharyngeal swabs for each run. These swab samples were collected from the routine diagnostics at the University Hospital Basel. The pooled eSwab solutions were split (1mL per tube) for each of the DNA extraction kits and centrifuged at 5000 g for 15 min. The supernatant is removed, and the pellets are used for DNA extraction. The sequencing run was performed three times with three technical replicates (*n* = 9). According to the Swiss Human Research Act, fully anonymized samples can be used for assay validation and development purposes. No patient-related data were used and all human reads were removed. We have used the pooled swab samples for the sequencing quality assessment and these samples were not analyzed for taxonomy identification and AMR gene prediction.

*DNA extraction kits.* After compiling the microbial communities, we next compared four DNA extraction kits. We selected the DNA extraction kits based on the following criteria: (i) The kit should be suitable for downstream sequencing; (ii) the DNA extraction process should have the potential to be automated; (iii) acceptable DNA quantity and quality from swab samples for ONT sequencing. We selected the QIAamp DNA Mini kit (DM, enzymatic lysis with Lysozyme (20 mg/mL) and Proteinase K (20 mg/mL), the QIAamp PowerFecal Pro DNA kit (PF, chemical and mechanical lysis with bead beating, using the inhibitor removal protocol), the Maxwell RSC Cultured Cells kit from Promega (CC, enzymatic lysis with Lysozyme (20 mg/mL)), and the Maxwell RSC Buccal Swab DNA kit from Promega (BS, enzymatic lysis with Proteinase K). For the QIAamp PowerFecal Pro DNA kit, the bead beating was performed at 25 Hz for 5 min using the Qiagen TissueLyser II (Germany). All microbial community samples were processed with these four kits according to the manufacturer’s instructions. Finally, we measured the extracted DNA quantity using the Thermo Fisher Scientific (Germany) Qubit 4™ Fluorometer with 1x dsDNA HS Assay Kit™ and quality using NanoDrop.

*Library preparation and sequencing.* We used the Rapid Barcoding Kit (SQK-RBK004) for ONT library preparation, given its faster TAT and better recovery of plasmid sequences^35^. We prepared the libraries according to the manufacturer’s instructions in the protocol version RBK_9054_v2_revR_14Aug2019. We multiplexed the libraries from Qiagen and Promega separately in different flow cells. The Zymo Mock Community, ESKAPE Mock, and swab samples were sequenced separately. Each sequencing run had six samples multiplexed together. We normalized the starting DNA concentration according to the sample types to avoid over or under-sequencing. All libraries were sequenced in R9.4.1 flow cells in GridION for a default of 72 h.

*Base-calling and demultiplexing.* The generated fast5 files were base-called with the Guppy base caller (v5.0.16) with the high accuracy model using the inbuilt MinKNOW (v21.05.25) software on a GridION. We performed the demultiplexing using the MinKNOW software. We transferred the data to the computing clusters sciCORE (http://scicore.unibas.ch/) scientific computing core facility at the University of Basel and ScienceCluster at the University of Zurich for downstream bioinformatic data analysis.

**Fig. 2:**
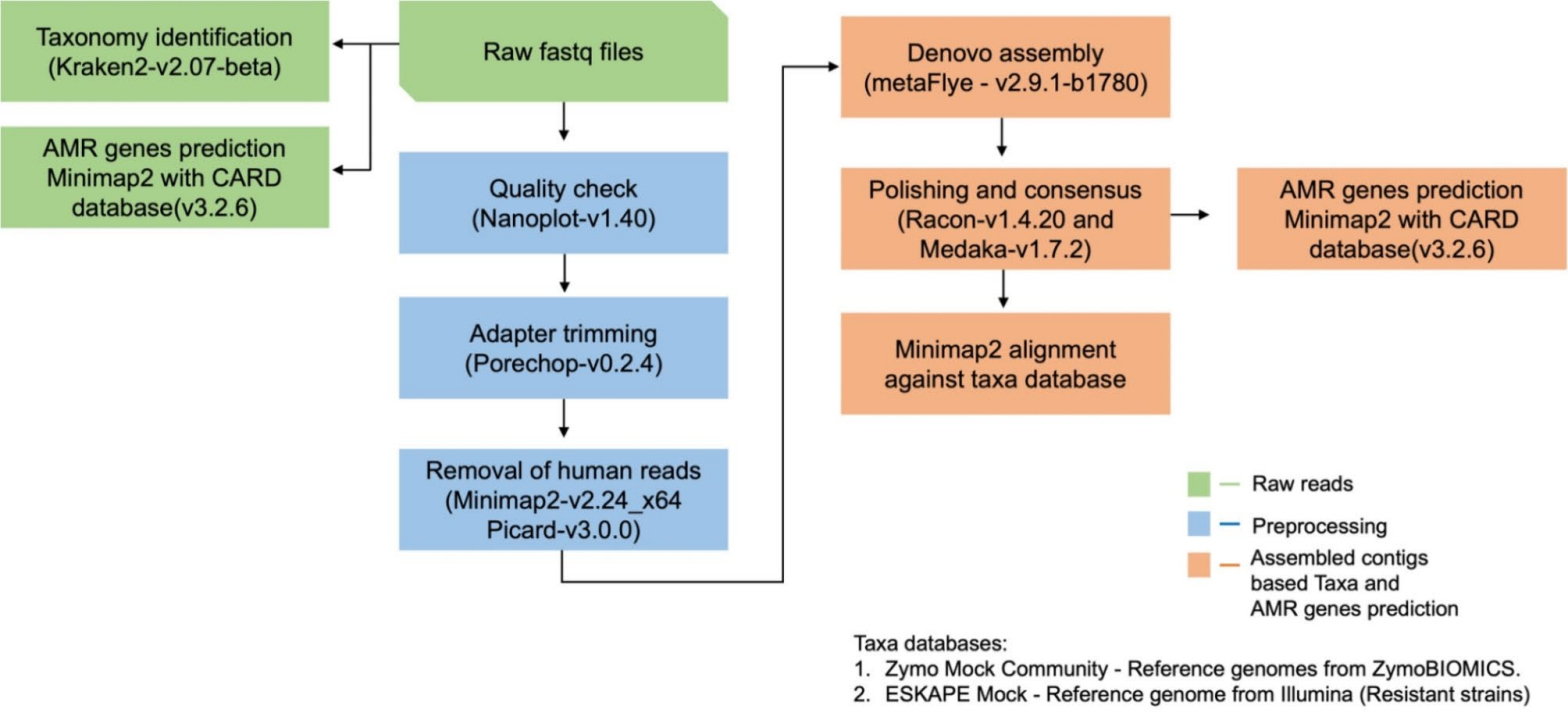
Bioinformatics data analysis workflow. The green boxes show the analysis performed on the raw reads, the blue boxes show the preprocessing steps, and the orange boxes show the de novo assembly and the analysis carried out on the de novo assembled contigs.

*Bioinformatics analysis.* For the bioinformatic process, we have used the default settings of the tools unless mentioned otherwise. We first performed a quality control analysis using the sequencing summary file in NanoPlot (v1.40)^48^. The raw fastq files were concatenated and renamed according to the sample name. We used Porechop (v0.2.4)(http://github.com/rrwick/Porechop?tab=readme-ov-file) for adapter trimming after which the adapter-trimmed reads were aligned against the human genome (Hg38.p13) using Minimap2 (v2.24_x64)^49^ with map-ont option and host reads were removed using Samtools (v1.6)^49^ and Picard (v3.0.0)(http://broadinstitute.github.io/picard/) was used to convert bam files to fastq files. Next, we performed a *de-novo* assembly of the non-human reads using Flye (v2.9.1-b1780)^50^ with nano-raw option in meta mode. The assembled contigs were polished (1x) using Racon (v1.4.20)(https://github.com/isovic/racon) and a consensus was built using Medaka (v1.7.2)(https://github.com/nanoporetech/medaka). We performed taxonomy identification at the read level using Kraken2(v2.0.7-beta)^51^ with a minikraken2_v2_201904 database for all the mock communities, owing to the less complexity of the mock samples. The default kmer length of k-35 was used. Next, we mapped the assembled contigs from the Zymo Mock Community samples against the reference provided by ZymoBIOMICS available at https://zymoresearch.eu/collections/zymobiomics-microbial-community-standards/products/zymobiomics-microbial-community-standard. ZymoBIOMICS has updated the isolate sequences used in the microbial community standard preparation, the latest community sequences deposited by ZymoBIOMICS does not match the references used in this manuscript. Then, we also mapped the assembled contigs from the ESKAPE mock against the Illumina-generated contigs for the resistant strains. We aligned the contigs using Minimap2 with full genome/assembly alignment mode with asm5 (5% sequence divergence). The generated paf files were processed using the pafr (v0.0.2) (https://dwinter.github.io/pafr/) package. Bases aligned with an exact match to the reference genomes, and which were of primary alignment, with mapping quality score (MAPQ) greater than 40 were considered. The bioinformatics analysis steps were visualized in a flowchart (Fig. 2).

**Fig. 3:**
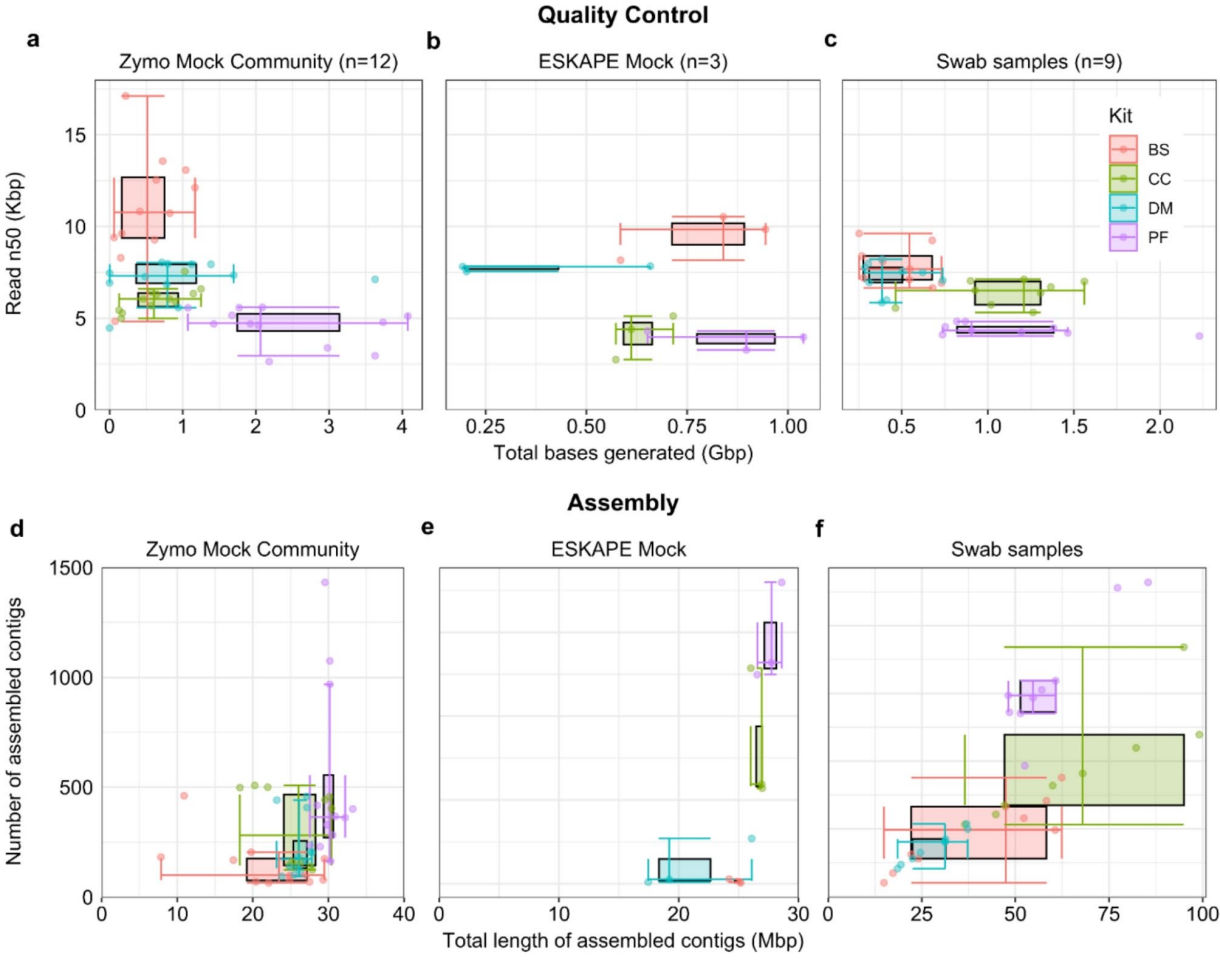
Sequencing quality of different extraction kits and microbial communities. Quality Control and *de novo* assembly summary from NanoPlot and Flye for the three sample types from four different DNA extraction kits. **a**-**c** represents a double box plot plotted total bases generated (in gigabases) against the read length N50 (in kilobases). **d**-**f** Double box plot for the total length of assembled contigs (in megabases) against the number of assembled contigs. Each color represents one DNA extraction kit. BS, Buccal Swab; CC, Cultured Cells; DM, QIAamp DNA Mini; and PF, QIAamp PowerFecal Pro DNA. The number of tested samples is provided in the brackets, which represent the technical and biological replicates of the Zymo Mock Community, the pooled swab samples, and the technical replicates of the ESKAPE Mock.

We predicted resistance genes for the ESKAPE Mock using Minimap2 alignment against the Comprehensive Antibiotic Resistance Database (CARD, v3.2.6)^52^ on the raw reads and assembled contigs with the same Minimap2 settings as mentioned before. The gene length coverage was obtained using Samtools(v1.15.1)^49^ coverage for the respective AMR genes. There is no specific gene length coverage cutoff used to filter out genes, as the question is to detect the presence or absence of the known AMR genes from the ESKAPE mock to highlight the differences from the kits. The sequencing summary files generated during the respective sequencing runs were used as input to determine the time of detection, i.e., the first time the read containing the specific AMR gene from the ESKAPE Mock appeared during the sequencing run across the four different DNA extraction kits. We used RStudio to generate the figures (v4.3.0). The code for double boxplot (Fig. 3) was obtained from https://stackoverflow.com/questions/46068074/double-box-plots-in-ggplot2.

We used the ChatGPT-4 to improve the grammar and readability of the manuscript, while the original manuscript was written by the authors.

## Results

### DNA concentration

We measured the quality and quantity of the extracted DNA for all extraction kits. Promega kits resulted in higher DNA concentrations compared to the Qiagen kits. Detailed values of DNA quantity and quality for each of the sample types are provided in supplementary Table S1. The DNA concentration of the pooled swab samples represents both host and microbial DNA. The mean DNA concentration and A_260/280_ ratio are provided in Table 1.

**Table 1:** Mean DNA concentration.

Sample type	Buccal Swab - Promega (BS, ng/µL)	Cultured Cells - Promega (CC, ng/µL)	QIAamp DNA Mini - Qiagen (DM, ng/µL)	QIAamp Power Fecal Pro - Qiagen (PF, ng/µL)
Zymo Mock Community (*n* = 12) A_260/280_	45.1 ± 20.0 2.0 ± 0.1	38.6 ± 7.7 2.0 ± 0.0	12.6 ± 5.1 2.1 ± 0.1	10.1 ± 2.1 2.0 ± 0.2
ESKAPE Mock (*n* = 3) A_260/280_	268.6 ± 96.8 2.0 ± 0.1	59.2 ± 24.0 2.0 ± 0.1	40.0 ± 31.8 1.9 ± 0.0	33.7 ± 7.9 1.8 ± 0.0
Pooled swab samples (*n* = 9) A_260/280_	245.8 ± 89.1 1.8 ± 0.0	87.9 ± 18.1 1.9 ± 0.0	49.2 ± 13.3 1.9 ± 0.0	44.6 ± 3.3 1.9 ± 0.0

The mean and standard deviations of DNA concentrations and the A_260/280_ ratio resulting from the four different DNA extraction kits are shown. The number of samples is indicated in closed brackets.

### Sequencing quality control and de novo assembly

First, we assessed sequencing quality using the sequencing summary file obtained from the MinKNOW software for the four DNA extraction kits by the number of bases generated and the read length N50. From Fig. 3a, we observed that the number of bases generated is higher in the PF followed by the CC kit, the DM kit, and the BS kit in the Zymo Mock community. In the ESKAPE Mock community (Fig. 3b), the number of bases generated is higher in the PF kit followed by the BS kit, the CC kit, and the DM kit. In the pooled swab samples (Fig. 3c), the number of bases generated is higher in the PF kit followed by the CC kit, the BS kit, and the DM kit.

In addition, we also noted that the read length N50 is the longest for the BS kit followed by the DM kit, the CC kit, and the PF kit. For the swab samples, Fig. 3c is plotted after removing human reads. The alignment percentages to the host (Human) genome are provided in supplementary Table S3. The overall alignment to the host genome is ≤ 1%. After removing the host reads, we assembled the contigs. The assembly parameters like the total length of assembled contig and number of contigs from Flye summary were obtained. The outputs across all DNA extraction kits were compared. The theoretical total or cumulative length of all assembled bacterial genomes in the Zymo Mock Community is 30.94 Mbp. We have observed that *de novo* assemblies resulting from the PF kit have generated a mean of 30.14 Mbp cumulative length, followed by the DM with 26 Mbp, the CC kit with 25.67 Mbp, and the BS kit with 21.78 Mbp (Fig. 3d). For the ESKAPE Mock the expected theoretical total length of assembled contigs would be 52.38 Mbp. After the assembly, we obtained a mean total assembled contig length of 27.63 Mbp for the PF kit followed by the CC kit (26.64 Mbp), the CC kit (24.83 Mbp), and the DM kit 26.64 Mbp (Fig. 3e). Supplementary Tables S2 and S4 provide the quality and assembly metrics. Since the theoretical assembled length for the biological swab samples is not known, we have reported the observed longest mean assembled contig length in the CC kit (71.2Mbp), followed by the PF kit (59.5 Mbp), the BS kit (39.9 Mbp), and the DM kit (28.1 Mbp).

**Fig. 4:**
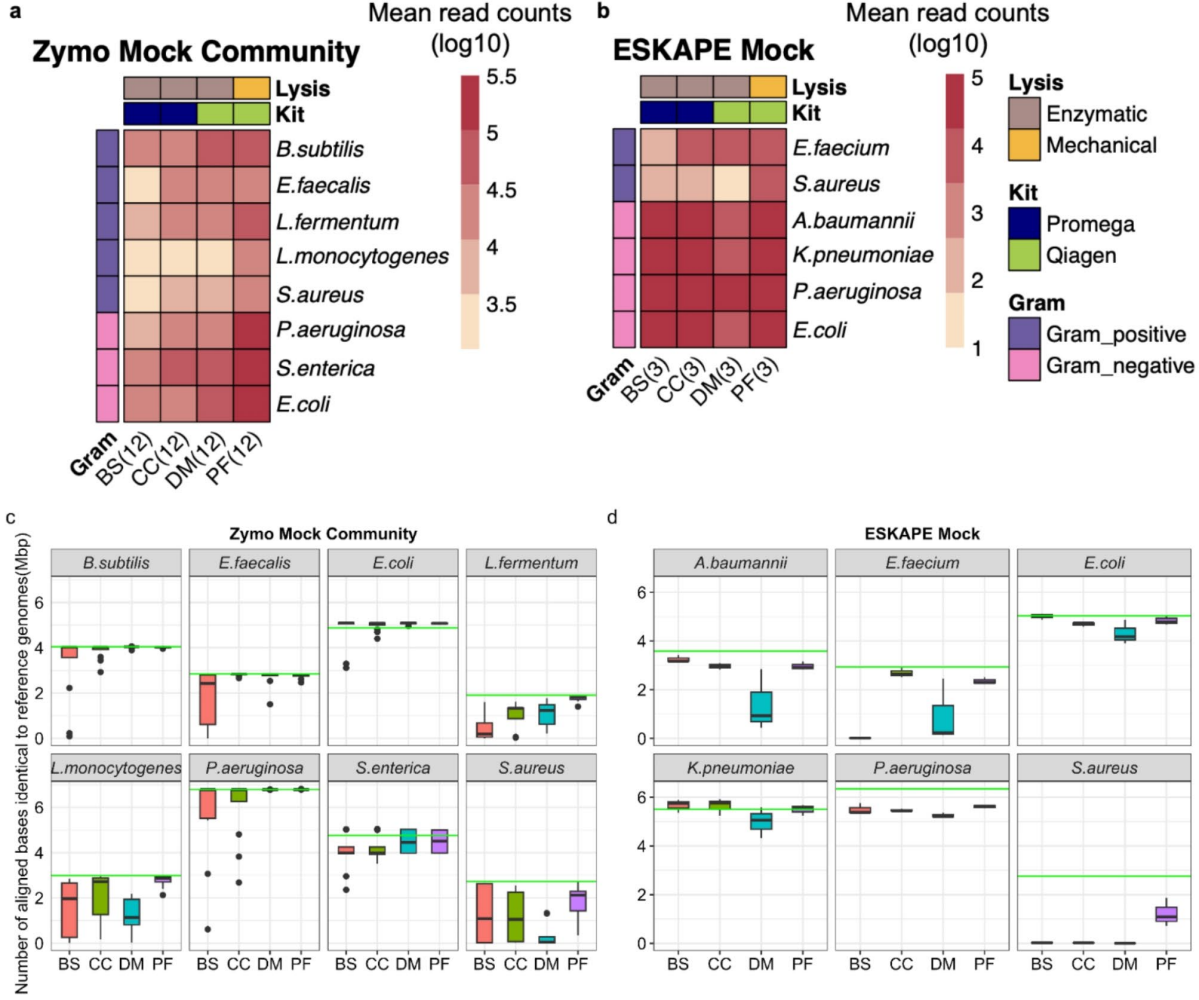
Performance of taxonomy identification. **a** and **b** Heatmaps with the mean read counts (log10 scale) from the raw reads for the Zymo Mock Community and the ESKAPE Mock using Kraken2. Kits used were BS, Buccal Swab; CC, Cultured Cells; DM, QIAamp DNA Mini; and PF, QIAamp PowerFecal Pro DNA. The total number of tested samples is provided inside the brackets(x-axis) which represent the technical and biological replicates for the Zymo Mock Community and the technical replicates for the ESKAPE Mock. **c** and **d** Assembled contigs alignment to the reference genomes of the Zymo Mock Community and ESKAPE Mock. The x-axis represents the different DNA extraction kits, and the y-axis represents the number of identical bases aligned to the respective reference genomes. The horizontal green line indicates the actual genome size of the respective bacterial species.

Taxonomy classification. We carried out the taxonomy identification on raw reads and assembled contigs on the mock communities. Figure 4a,b shows the mean read counts from Kraken2 for Zymo Mock Community, and ESKAPE Mock, respectively (Fig. 4a,b). We observed, differences in the annotated mean read counts within the four different extraction kits for Gram-positive and Gram-negative species and across the kits. The overall percentage of false positive bacterial phylum detected for the reads generated with all the kits is ≤ 0.02%. Supplementary Table S5.1 has the read counts associated with the false positive bacterial phylum and supplementary Table S5.2 has the read counts associated with the Bacteria, Viruses, Archaea, and Eukaryotes for all the kits.

Taxonomy classification on the reads by the kmer-based Kraken with minikraken2 database can yield false positives due to misclassification at the species level^53^ due to evolutionary relatedness. Therefore, we also mapped the assembled contigs against the reference genome sequences for Zymo Mock Community and ESKAPE Mock.

Figure 4c represents assembled contigs alignments to the reference genomes of the Zymo Mock Community and Fig. 4d represents the assembled contigs alignment to the reference Illumina genomes of resistant ESKAPE strains. The contigs assembled from the reads of the enzymatic kits have lower aligned bases for Gram-positive species, especially in the ESKAPE Mock compared to the contigs assembled with the reads generated from the PF kit.

### Resistance prediction

**Fig. 5:**
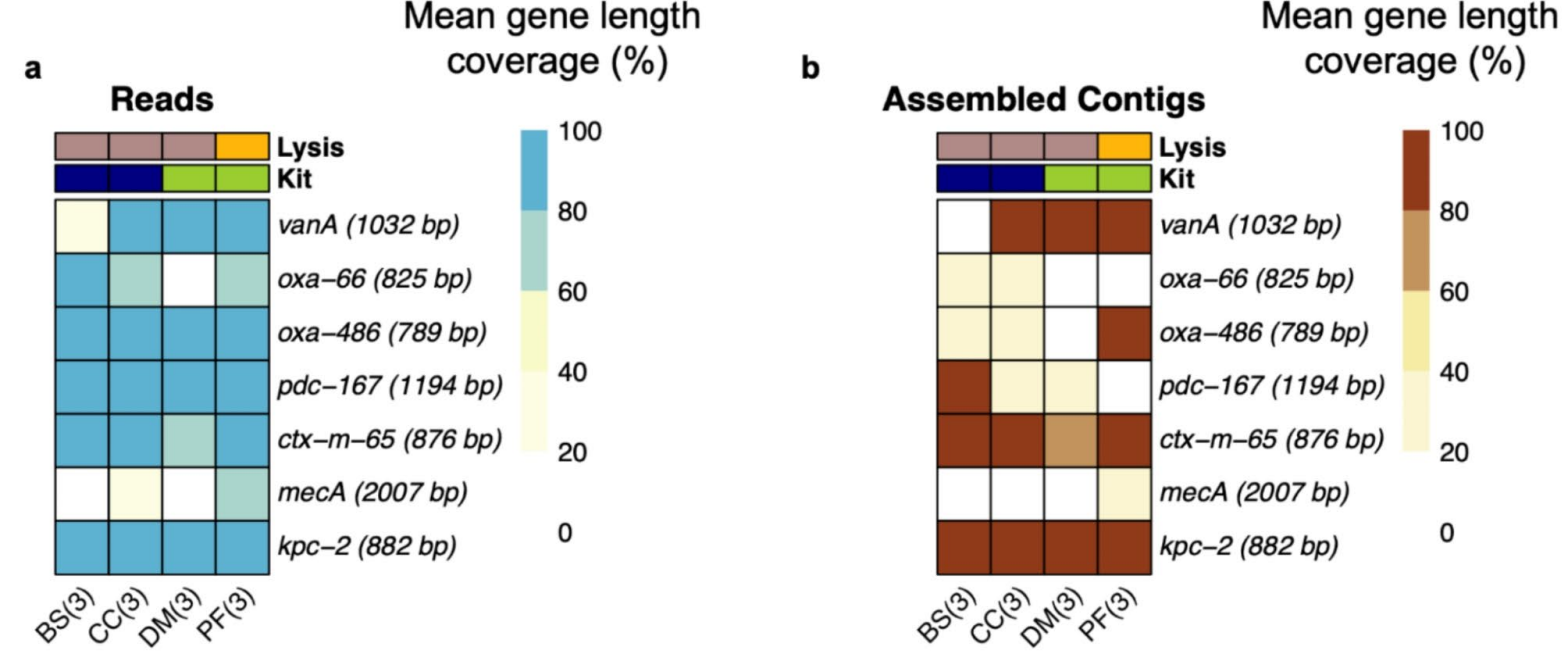
AMR genes prediction. The figure shows the mean gene length coverage percentage on raw reads (left) and assembled contigs (right) for the ESKAPE Mock sample. Kits used were BS, Buccal Swab; CC, Cultured Cells; DM, QIAamp DNA Mini; and PF, QIAamp PowerFecal Pro DNA. The total number of tested samples is given inside the brackets (x-axis), which represent the technical replicates of the ESKAPE Mock. The respective gene lengths of the detected AMR genes are shown in brackets (y-axis). The white box represents the absence of AMR genes (0% gene length coverage).

AMR gene prediction is performed with the Minimap2 alignment of the raw reads and assembled contigs against the CARD database. The reads generated from the PF kit identified all the relevant AMR genes in the ESKAPE mock community. Figure 5a,b show the mean gene length coverage (%) for all the kits as heatmaps on the raw reads and assembled contigs. Based on the reads generated with the DM and BS kits, we were not able to detect all resistance genes, especially from Gram-positive species *E. faecium* and *S. aureus.*

The CC kit detected resistance genes from the Gram-negative species and *vanA* from Gram-positive *E. faecium*, but it failed to detect *mecA* from *S. aureus* in two out of three biological replicates also on the contigs level CC kit was not able to detect mecA from *S. aureus* while the DM and BS kits failed to detect mecA from *S. aureus* in all the biological replicates. The DM kit detected resistance gene vanA from Gram-positive *E. faecium* but failed to detect *oxa-66* from Gram-negative *A. baumannii* and *mecA* from Gram-positive *S. aureus*. While the reads generated from the PF kit detected the *oxa-66* gene on the read level, it failed to detect the gene from the assembled contigs. The complete list of genes obtained from the CARD database alignment for the reads and contigs is provided in supplementary Tables S6.1 and S6.2.

*Time to detect AMR genes.* We calculated the time to detect the predicted AMR genes across the different kits using the sequencing summary files obtained from the respective sequencing run folders. Table 2 shows the median time of detection for the reads containing the expected AMR genes and interquartile range for the first 24 h of sequencing.

The reads generated from the PF kit detected all the expected AMR genes from the ESKAPE Mock with a maximum median sequencing time of 1.9 h (Table 2), while the overall TAT with the PF kit was 10.5 h with DNA extraction (1.5 h), library preparation(2.5 h for six libraries), sequencing (2.5 h) and AMR genes and taxa identification on the read level (~ 4 h).

**Table 2:** Median time in hours(h) for the expected AMR genes detected in the 24 h of sequencing.

DNA extraction kits	AMR genes	Median time (h)	Inter quartile range (h)
BS	*ctx-m-65*	1.8	0.5
	*kpc-2*	1.0	0.3
	*oxa-66*	2.1	8.1
	*pdc-167*	2.0	1.2
	*oxa-486*	0.4	0.5
	*vanA*	0	0
	*mecA*	0	0
CC	*ctx-m-65*	2.4	0.9
	*kpc-2*	0.7	0.4
	*oxa-66*	4.8	2.7
	*pdc-167*	0.9	0.4
	*oxa-486*	0.8	0.3
	*vanA*	2.8	1.6
	*mecA*	0	7.4
DM	*ctx-m-65*	1.9	4.6
	*kpc-2*	2.0	1.6
	*oxa-66*	0	0
	*pdc-167*	3.5	4.8
	*oxa-486*	2.0	0.5
	*vanA*	1.2	0.8
	*mecA*	0	0
PF	*ctx-m-65*	0.7	0.2
	*kpc-2*	1.2	0.4
	*oxa-66*	0.8	1.7
	*pdc-167*	0.4	0.1
	*oxa-486*	0.6	0.5
	*vanA*	1.8	1.4
	*mecA*	1.9	1.8

The median time for the first occurrence of the read with the expected AMR gene is reported. *BS* Buccal Swab; *CC* Culture Cells Kit; *DM* QIAamp DNA Mini; *PF* QIAamp PowerFecal Pro DNA.

## Discussion

In this study, we evaluated four different DNA extraction kits in combination with two different bacterial mock communities namely the Zymo Mock Community, an *in-house* ESKAPE Mock, and pooled swab samples for rapid long-read shotgun metagenomics with ONT. The biases arising from the DNA extraction kits in microbiota composition analysis are widely discussed in the scientific community^40,43,54–56^, emphasizing the importance of the selection of suitable DNA extraction kits for different sample types. With the advent of long-read sequencing technologies, efforts were made to extract high molecular weight DNA from various sample types to harness the power of these long-read sequencing platforms^57,58^.

From the DNA quality results we have observed that the A_260/280_ ratios are found to be in the acceptable range of 1.8-2.0 for all kits. It should also be noted that the A260/230 ratio (supplementary Table S1) is highly dependent on the kit chemistries, and DNA concentration. Additionally, we also used nuclease-free water to blank the NanoDrop because the exact composition of the DNA elution buffer is not known. This could potentially contribute to the lower A260/230ratios(https://assets.thermofisher.com/TFS-Assets/CAD/Product-Bulletins/TN52646-E-0215M-NucleicAcid.pdf*).* The Promega kits with magnetic bead-based purification have resulted in higher DNA yield compared to the Qiagen kits with spin column-based purification.

Our study has shown that the enzymatic lysis-based kits generated longer read length N50 compared to the kit with chemical and mechanical lysis. This result is expected as the bead-beating process in the PF kit shears the DNA. Owing to the shorter read length N50 the PF kit also generated higher data yield compared to the other enzymatic lysis-based kits as ONT preferentially sequences shorter DNA fragments over the longer fragments, due to the faster translocation speed of the shorter fragments through the nanopores^59,60^.

Even with a shorter read length N50, the PF kit achieved the near theoretical total assembled contig length in the Zymo Mock Community and the longest assembly length in the in-house ESKAPE Mock^61^. Of note, none of the kits matched the theoretical total assembled contig length in the in-house ESKAPE Mock, which could arise from the *de novo* assembler collapsing the sensitive and resistant strains of the same species together into the same contig^62,63^.

Using shotgun metagenomics with ONT sequencing in routine diagnostics needs to answer two key questions: (i) Which bacterial species is present (identification) and (ii) what AMR genes can be detected and are functional^64^? Taxonomic analysis on the raw reads using Kraken2 revealed that the PF kit with chemical and mechanical lysis performed equally well for detecting the Gram-positive and -negative species. A similar finding has also been observed when the *de novo* assembled contigs are aligned against the reference genomes. The PF kit with chemical and mechanical lysis is a robust kit for the comprehensive detection of Gram-positive and -negative species in an unbiased manner from complex sample types. The usage of bead-beating-based DNA extraction is also recommended for shotgun metagenomics studies by the STROBE guidance^65,66^ for extracting DNA from bacterial species, which are difficult to lyse, in any complex samples. The reduced ability of the enzymatic lysis-based kits to detect Gram-positive species could be due to the presence of a thick peptidoglycan layer in the cell wall, which resists the enzymatic lysis^67^.

One could also argue that the PF kit has generated more bases than the other kit from the sequencing runs and resulted in more reads and aligned bases for the respective mock communities (Fig. 4a-d). However, when we compare within each kit there are differences between the reads and bases assigned to Gram-positive and Gram-negative species. Since the composition of the mock communities is known, the observed variability in the distribution of the reads (Fig. 4a-b) across different species from the DNA extraction kits potentially indicated an extraction bias arising from the DNA extraction chemistries. While we used the kmer-based Kraken2 for taxonomy identification due to its rapid computation, there are potential limitations of using Kraken2, such as the occurrence of false positive species and difficulty in estimating quantitative abundances, which are recognized issues in the community^68,69^. Since the taxa composition of the Zymo Mock community and ESKAPE Mock are known, we have not shown the false positive species detected by Kraken2 for the DNA extraction kit comparison.

Theoretically, if the kits show effective lysis against both Gram-positive and Gram-negative species the distribution of the aligned bases should be equal in accordance with the respective species and their genome sizes, but we have observed variation in the distribution of aligned bases, within each kit (Fig. 4c-d). This is a possible indication of Gram bias. So, it is convincing that kits without mechanical lysis have a reduced ability to extract tough-to-lyse Gram-positive species from mixed samples.

The differences observed between the aligned base counts (Fig. 4c-d) for the species, *S. aureus*,* P. aeruginosa*,* and E. coli* which are present in both the Zymo Mock Community and ESKAPE Mock could be due to the cryopreservation^70^, inactivation process, and storage solutions involved in the manufacturing of Zymo Mock Community. These processes may have led to compromised cell wall integrity, which in turn increased the susceptibility of Gram-positive and Gram-negative species to lysis in the Zymo Mock compared to the ESKAPE Mock, which is prepared with fresh pure cultures^71^.

The taxonomic and AMR identification for the pooled swab samples is not discussed as the composition for these samples is not known for a direct comparison to determine the extraction bias arising from the different DNA extraction kits. Also, we did not include negative controls during the sequencing runs with the swab samples to differentiate the true hits from contamination, which would have made the conclusions from the pooled swab samples possibly biased. However, we have uploaded the long-read sequencing data generated from the swab samples without any host reads into the European Nucleotide Archive (ENA).

For AMR gene prediction our data showed that raw reads generated from the PF kit can identify all relevant AMR genes from the Gram-positive and -negative species in the ESKAPE Mock.

Differences are observed in AMR prediction for the reads and assemblies (Fig. 5a,b). This could potentially be due to the uneven coverage of shotgun metagenomic sequencing, leading to the potential misassembly of the reads into contigs and missing AMR genes^72^. On the other hand, AMR prediction directly from the reads may improve AMR prediction, but care should be taken to assess the results as read mapping may result in hits to numerous gene variants and therefore false positives^73,74^. This could be one of the possibilities for observing other gene variants of the *oxa*, *ctx-m*,* kpc*, and *pdc* as the result of the multi-mapping of the reads against the CARD database which are provided in supplementary Tables S6.1 and S6.2. The inability of some kits (BS, DM, and CC) to detect *mecA* and *vanA* from the ESKAPE Mock, would be attributed to the ineffective lysis of the Gram-positive species, potentially leading to uneven to low coverage of the genome and failure to detect those genes.

We showed that long-read sequencing with ONT provides rapid detection of AMR genes within a median sequencing time of approximately 1.9 h of sequencing possible with the PF kit. Several studies involving complex clinical samples with low microbial biomass reported a median of 50 min to 6 h for detection^75,76^. The short time to detection in the in-house ESKAPE Mock sample is likely due to the sample mock being a predefined pure microbial culture, without the presence of the host and other commensals. A large validation study with clinical samples would be needed to assess the complete TAT from sample collection to AMR prediction for clinical intervention with antibiotics therapy. It should also be noted that the presence of AMR genes in the sample detected by the metagenomic sequencing would be an indication of the potential presence of resistant pathogens and not directly translate to a resistant phenotype, as it is also dependent on the gene level expression^77^. In addition, also for reliable detection of an AMR gene, multiple reads with a threshold would need to be determined from clinical samples.

While the current study evaluates DNA extraction kits for long-read metagenomics using mock communities, the exclusion of negative controls introduces a limitation. The controlled nature of this study, with predefined and well-characterized microbial compositions, minimizes the risk of misidentifying contaminants, reducing the immediate necessity for negative controls. However, in clinical settings where bacterial compositions are unknown and environmental contamination can obscure the detection of clinically relevant organisms, negative controls become crucial. The inclusion of negative controls will be essential for validating findings and ensuring the reliability of both taxonomic and AMR gene detection, particularly in low-biomass clinical samples^78^.

Considering the dynamic changes in the ONT pore chemistries and base-calling models, another limitation of our study would be that the evaluation was not performed on the newest R10.4 flow cells^79^ and the study results are tailored towards the library preparation using Rapid Barcoding Kit. Care should be exercised on the bioinformatics front, as the software tools and databases associated with taxonomy and AMR identification are dynamically developed and updated, and the results are restricted to the tools used in the manuscript. We have not benchmarked the bioinformatics tools used for the analysis, as it is beyond the scope of this study. However, our study systematically approached the challenge of DNA extraction bias for ONT-based shotgun metagenomics and can serve thereby as a blueprint for future validation studies.

## Conclusion

In this comparative study, four DNA extraction kits for long-read shotgun metagenomics on the ONT sequencing platform were tested. Notable distinctions in kit performance were observed in DNA extraction based on whether mechanical extraction was included or not. The spin column-based kits, such as Qiagen, yielded less DNA than magnetic bead-based kits like Promega but were still suitable for sequencing due to lower DNA input requirements for the library preparation using the Rapid Barcoding Kit. The QIAamp PowerFecal Pro DNA kit, which employs a bead beating process, resulted in shorter read lengths, but achieved near theoretical assembled contig lengths, underscoring its robustness in detecting both Gram-positive and -negative bacterial species and AMR genes associated with the resistant strains in the ESKAPE Mock. Furthermore, ONT facilitates the rapid identification of the AMR genes, with a median detection time of approximately 1.9 h. Thus, long-read shotgun metagenomics using ONT sequencing with Rapid Barcoding Kit and DNA extraction kits, especially those with mechanical extraction like QIAamp PowerFecal Pro DNA kit, demonstrate robustness for bacterial species and AMR gene detection from complex samples.

## Electronic supplementary material

Below is the link to the electronic supplementary material.

Supplementary Material 1

Supplementary Material 2

Supplementary Material 3

Supplementary Material 4

Supplementary Material 5

Supplementary Material 6

Supplementary Material 7

Supplementary Material 8

Supplementary Material 9

## Data Availability

The adapter and host removed long-read sequenced raw fastq files, have been deposited in the European Nucleotide Archive (ENA) with accession id: PRJEB75510 and will be made public upon acceptance of the manuscript.
