# Supplementary material for: Evaluation of DNA extraction kits for long-read shotgun metagenomics using Oxford Nanopore sequencing for rapid taxonomic and antimicrobial resistance detection
Source: Sci Rep. 2024 Nov 27;14:29531. doi: 10.1038/s41598-024-80660-3 (PMC11603047; doi:10.1038/s41598-024-80660-3)
Supplement: Supplementary file 1 — Supplementary Material 1 [file 41598_2024_80660_MOESM1_ESM.docx]

**Appendix**

**Clinical samples:** Collected from symptomatic patients for diagnostic testing.

**Screening samples:** Collected for infection control and prevention to identify potential asymptomatic carriers.

**Read length N50:** The sequenced read length where 50% of the sequenced bases are contained within reads of this length, or longer.

**Assembly N50**: Defined by the length of the shortest contig for which longer and equal length contigs cover at least 50 % of the assembly. [[80](https://paperpile.com/c/kyWzcZ/0Tb6)]

***ctx-m-65:*** Extended-Spectrum β-Lactamase (ESBL).

***oxa-66***: Beta-lactamase - Carbapeneamase.

***oxa-23***: Beta-lactamase - Carbapeneamase.

***oxa-486***: oxa-50 like beta-lactamase - Carbapenemase.

***pdc-167***: Pseudomonas Derived Cephalosporinase (PDC) beta-lactamase.

***vanA***: Glycopeptide resistance gene cluster, vanA ligase.

***mecA***: Methicillin-resistant penicillin binding protein 2(PBP2).
